# Perinatal protein restriction with postnatal catch-up growth leads to elevated p66Shc and mitochondrial dysfunction in the adult rat liver

**DOI:** 10.1530/REP-19-0188

**Published:** 2019-11-05

**Authors:** Shelby L Oke, Gurjeev Sohi, Daniel B Hardy

**Affiliations:** 1 The Children’s Health Research Institute, London, Ontario, Canada; 2 Lawson Health Research Institute, London, Ontario, Canada; 3 Department of Obstetrics and Gynaecology, London, Ontario, Canada; 4 Department of Physiology and Pharmacology, London, Ontario, Canada; 5 The University of Western Ontario, London, Ontario, Canada

## Abstract

Epidemiological data suggest an inverse relationship between birth weight and long-term metabolic deficits, which is exacerbated by postnatal catch-up growth. We have previously demonstrated that rat offspring subject to maternal protein restriction (MPR) followed by catch-up growth exhibit impaired hepatic function and ER stress. Given that mitochondrial dysfunction is associated with various metabolic pathologies, we hypothesized that altered expression of p66Shc, a gatekeeper of oxidative stress and mitochondrial function, contributes to the hepatic defects observed in MPR offspring. To test this hypothesis, pregnant Wistar rats were fed a control (20% protein) diet or an isocaloric low protein (8%; LP) diet throughout gestation. Offspring born to control dams received a control diet in postnatal life, while MPR offspring remained on a LP diet (LP1) or received a control diet post weaning (LP2) or at birth (LP3). At four months, LP2 offspring exhibited increased protein abundance of both p66Shc and the cis-trans isomerase PIN1. This was further associated with aberrant markers of oxidative stress (i.e. elevated 4-HNE, SOD1 and SOD2, decreased catalase) and aerobic metabolism (i.e., increased phospho-PDH and LDHa, decreased complex II, citrate synthase and TFAM). We further demonstrated that tunicamycin-induced ER stress in HepG2 cells led to increased p66Shc protein abundance, suggesting that ER stress may underlie the programmed effects observed *in vivo*. In summary, because these defects are exclusive to adult LP2 offspring, it is possible that a low protein diet during perinatal life, a period of liver plasticity, followed by catch-up growth is detrimental to long-term mitochondrial function.

## Introduction

Fetal undernutrition gives rise to intrauterine growth restriction (IUGR), a condition characterized by low birth weight and reduced organ growth. Epidemiological studies have established an inverse relationship between birth weight and long-term metabolic health, as individuals exposed to undernutrition *in utero* exhibit high rates of the metabolic syndrome in adult life (Ravelli *et al.* 1998, Roseboom *et al.* 2001). This relationship is further exacerbated by postnatal catch-up growth, whereby the affected offspring undergo rapid weight gain during critical stages of growth and development. Collectively, this underlies Barker’s ‘thrifty phenotype’ hypothesis, which states that an adverse intrauterine environment will cause permanent alterations to physiological processes in anticipation of a similarly hostile postnatal environment (Hales & Barker 2001). When a mismatch in nutrient availability occurs between the pre- and postnatal environments, these adaptations become maladaptive and pose risk for development of the metabolic syndrome (Hales & Barker 2001, Ozanne & Hales 2004, Bieswal *et al.* 2006, Sohi *et al.* 2011). While animal studies have revealed the contributions of postnatal catch-up growth to long-term dysmetabolism, the molecular basis of the relationship between birth weight and postpartum development remains poorly understood.

There is strong evidence to suggest that the composition of maternal diet during pregnancy plays a role in fetal health. Given that amino acids are essential for fetal growth and development (Battaglia & Meschia 1978, Crosby 1991), the maternal protein restriction (MPR) model of undernutrition has been widely utilized in rodents to investigate the role of protein availability on postnatal outcomes. We and others have demonstrated that MPR offspring are low birth weight and exhibit asymmetrical organ growth, with the fetal liver becoming selectively compromised at the expense of other organs such as the lungs and the brain (Desai & Hales 1997, Sohi *et al.* 2011, 2013). Not surprisingly, MPR offspring undergo hepatic and whole-body catch-up growth when introduced to a normal protein diet at birth or weaning (Ozanne & Hales 2004, Sohi *et al.* 2011). Our laboratory has established that these ‘recuperated’ offspring exhibit signs of impaired hepatic function at adulthood, including hypercholesterolemia, glucose intolerance (e.g., increased gluconeogenesis) and accelerated drug catabolism due to differential abundance of hepatic enzymes (Sohi *et al.* 2011, 2014, Vo *et al.* 2013). Conversely, MPR offspring without catch-up growth have normal cholesterol levels and drug metabolism later in life, but to date, the mechanisms underlying rapid catch-up growth and dysmetabolism are unclear (Sohi *et al.* 2011, 2014).

Mitochondria are intracellular energy producers that are largely responsible in regulating metabolism and oxidative stress. Moreover, impaired mitochondrial function is associated with various metabolic pathologies (Petersen *et al.* 2003, Nojiri *et al.* 2006, Ozgen *et al.* 2012), and a variety of maternal insults have been shown to compromise mitochondrial function in IUGR offspring (Park *et al.* 2003, Moraes *et al.* 2014, Barra *et al.* 2017, Woodman *et al.* 2018). While these studies demonstrate the importance of the maternal nutrition in mediating mitochondrial function, it remains unknown whether these abnormalities occur directly due to the maternal insult or due to postnatal catch-up growth. For example, using a model of maternal nicotine exposure (MNE), we have previously shown that IUGR offspring have both impeded mitochondrial function and cardiac dysfunction exclusively after catch-up growth has occurred (Barra *et al.* 2017). This may also occur in nutrition-induced IUGR offspring (e.g. MPR); however, the critical windows of nutrient deprivation leading to impaired mitochondrial function and metabolic disease remain elusive.

Several studies have implicated the p66Shc adaptor protein in processes contributing to mitochondrial dysfunction and oxidative stress (Migliaccio *et al.* 1999, Orsini *et al.* 2004, Giorgio *et al.* 2005, Pinton *et al.* 2007, Trinei *et al.* 2013). Upon cellular stress, cytosolic p66Shc becomes phosphorylated at serine residue 36 (Ser36), followed by a subsequent conformational change that is initiated by the cis/trans isomerase PIN1 (Giorgio *et al.* 2005, Pinton *et al.* 2007). After dephosphorylation of Ser36 by PP2A phosphatase, p66Shc undergoes mitochondrial translocation to permit binding with cytochrome C. This interaction promotes increased production of ROS, as well as accelerated rates of mitochondrial-induced apoptosis and cellular senescence (Migliaccio *et al.* 1999, Giorgio *et al.* 2005). Studies have shown that activation of p66Shc also leads to compromised aerobic metabolism, which may further contribute to increased reactive oxygen species (ROS) production (Acin-Perez *et al.* 2010, Lone *et al.* 2018). While the mechanism by which this occurs is not yet clear, there is evidence to suggest that increased p66shc activation is linked to metabolic pathologies such as diabetes and coronary artery disease (Pagnin *et al.* 2005, Noda *et al.* 2010). Therefore, the overall objective of this study was to determine if MPR offspring exhibit hepatic mitochondrial dysfunction following postnatal catch-up growth, and to define if this was concomitant with elevated levels of p66Shc and oxidative stress in adulthood. Furthermore, given that increased p66Shc expression has been associated with endoplasmic reticulum (ER) stress (Zhang *et al.* 2013), and MPR offspring with catch-up growth exhibit hepatic ER stress (Sohi *et al.* 2013), we further anticipated that ER stress may mediate any increase in p66Shc expression.

## Materials and methods

### Animals and dietary regimes

**Figure 1:**
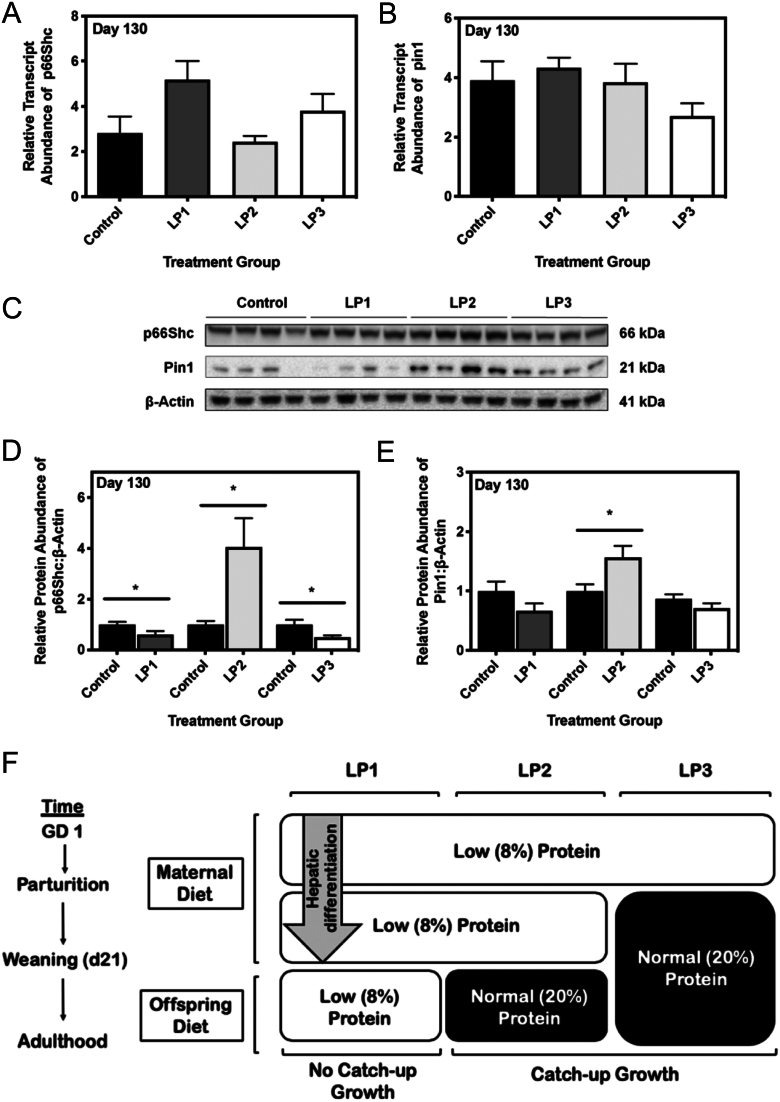
Maternal protein restriction (MPR) and postnatal catch-up growth together increase hepatic p66Shc and PIN1 in 4-month-old male offspring. (A) Percent protein composition and timing of nutritional intervention of each MPR diet. Transcript and protein abundances of p66Shc and Pin1 were determined via quantitative real-time PCR and Western immunoblotting, respectively. Relative transcript abundance of (B) p66Shc and (C) Pin1 were expressed as means normalized to the geometric mean of β-actin and GAPDH ± s.e.m. (n = 7–8/group). (D) Representative western immunoblots for specific targeted protein bands of control, LP1, LP2 and LP3 offspring as detected by primary antibodies for p66Shc and PIN1. Relative protein abundances of (E) p66Shc and (F) PIN1 at 4 months of age were expressed as means normalized to β-Actin ± s.e.m. (*n* = 7–8/group). All qRT-PCR data were analyzed using a one-way ANOVA and multiple comparisons test, while protein abundances were compared using a two-tailed unpaired Student’s *t*-test. *Significant difference (*P* < 0.05).

All procedures were performed according to guidelines set by the Canadian Council of Animal Care with approval from the Animal Care Committee at The University of Western Ontario. Male and female Wistar rats (250 g, 8–10 weeks old) were purchased from Charles River (La Salle, St. Constant, QC) and left to acclimatize to environmental conditions of the animal care facility for 3 weeks. Upon proestrus, female rats were placed in cages of male rats for mating, and pregnancy was confirmed the next morning via presence of sperm in the vaginal smear (gestational day 1). Pregnant dams were then fed either a normal (20% casein) protein diet or a low (8% casein, LP) protein diet for the remainder of gestation (Fig. 1A). Diets were made isocaloric with each other through the addition of carbohydrates (i.e. sucrose) to the LP diet as we have previously published (Sohi *et al.* 2015), and food was provided *ad libitum* throughout pregnancy. Despite the increased carbohydrate content, the LP diet is not considered to be high in carbohydrates as the addition of sucrose is modest (i.e., an increase of 13%) in comparison to decreased total protein (i.e., a decrease of 60%). At birth, litter size was reduced to eight animals by selecting pups with birth weight closest to the litter mean. Offspring born to control diet-fed mothers continued to feed off a control diet for the remainder of life, while offspring born to LP dams were assigned to one of three LP groups (Fig. 1A): low protein 1 (LP1), low protein 2 (LP2) or low protein 3 (LP3). LP1 offspring were fed a LP diet throughout life, while LP2 offspring were introduced to a normal protein diet at weaning (postnatal day 21). Given that hepatic differentiation in rats is not complete at birth (Gruppuso & Sanders 2016), the LP2 group of offspring allows us to examine the effects of protein restriction during the entire perinatal period. LP3 offspring received a control diet at birth and continued to feed on this diet for the remainder of life. Necropsy was performed at postnatal days (PND) 21 and 130 to examine the direct and indirect effects of MPR, respectively. As demonstrated by our studies and others, PND 130 was chosen given it is the time at which poor metabolic outcomes (e.g. glucose intolerance and hypercholesterolemia) are manifested in these low birth weight offspring (Chamson-Reig *et al.* 2009, Sohi *et al.* 2011). For the purposes of this study, male offspring were exclusively selected because female MPR offspring exhibit less metabolic deficits (e.g. normal cholesterol levels) and to avoid confounding effects presented by the female estrus cycle (Sohi *et al.* 2011). The right medial hepatic lobe was collected and immediately flash-frozen in liquid nitrogen, followed by storage at −80°C until further use.

### Cell culture and induction of ER stress

The HepG2 human hepatocellular carcinoma cell line was obtained from the American Type Culture Collection (ATCC) and cultured in 5% CO_2_/95% atmospheric air at 37°C. Cells were maintained in minimum essential medium (MEM; Gibco) supplemented with 10% fetal bovine serum (FBS; Gibco) and 1% penicillin-streptomycin solution (10,000 IU and 10,000 µg/mL, respectively; Fisher Scientific). For induction of ER stress, cells between passages 7 and 10 were seeded at a density of 2.0 × 10^5^ cells/well in six-well culture plates (Thermo Scientific) and allowed to proliferate for 24 h prior to treatment. Cells were then treated with 0.5, 1.0 and 2.0 µg/mL tunicamycin (BioShop Canada, Burlington, ON) for 1, 2 and 6 h. Additional cells were treated with a matching volume of DMSO as a vehicular control for 1, 2 and 6 h. Following treatment, cells were collected for protein isolation and western immunoblotting as described below.

### RNA isolation and real-time PCR analysis

Total RNA was isolated from PND 21 and 130 livers using the one-step method of Chomczynski and Sacchi (TRIzol; Invitrogen), followed by RT with a High-Capacity cDNA RT Kit (Applied Biosystems). Primer sets for gene targets of interest were designed using the National Center for Biotechnology Information and Ensembl genome browsers, followed by generation via Invitrogen Custom DNA Oligos (Table 1). Relative transcript abundance was determined via quantitative real-time PCR (qRT-PCR) as previously published (Barra *et al.* 2017). Values obtained for all gene targets of interest were normalized to geometric means of β-Actin and *GAPDH*, which were determined to be suitable housekeeping genes by using both the comparative ∆Ct method and algorithms from geNorm, Normfinder and BestKeeper (Vandesompele *et al.* 2002, Pfaffl *et al.* 2004). Relative transcript abundance was calculated for each primer set as determined by the comparative ∆Ct method.

**Table 1:** Forward and reverse sequences for primers used in analysis of mRNA targets via quantitative real-time PCR.

Gene	Forward sequence	Reverse sequence	GenBank/Reference
*Pin1*	CAGCTCAGGCCGTGTCTACTA	TCCGAGATTGGCTGTGCTTC	NM_001106701.2
*p66Shc*	TACTTGGTTCGGGTGAGTGC	GAGCAGGAAGTCCCGACAAA	NM_053517.2
*ND1*	CCGAGAACGCAACTCAGGTA	CCTAAGACACCACCAGCATGT	NM_001006972.1
β-Actin	CACAGCTGAGAGGGAAAT	TCAGCAATGCCTGGGTAC	NM_031144
*GAPDH*	GGATACTGAGAGCAAGAGAGAGG	TCCTGTTGTTATGGGGTCTGG	NM_017008.4

### Protein extraction and western immunoblot

Whole-cell protein lysates were isolated from liver segments and HepG2 cells as previously published (Barra *et al.* 2017). Protein loading samples were prepared for western immunoblotting using sample lysates, NuPAGE reducing agent (10×; Invitrogen), NuPAGE LDS sample buffer (4×; Invitrogen), and deionized water. Loading samples were heated at 70°C to denature proteins, followed by loading into wells of 4–12% Bis-Tris gels (Invitrogen). For liver samples, 20 µg of protein was loaded per well, while cell samples were loaded at 10–12 µg per well. Following separation by gel electrophoresis, proteins were transferred onto PVDF membranes (Thermo Scientific) at 100 volts for 2 h. Membranes were blocked in 1× Tris-buffered saline/Tween-20 (TBST) buffer with either 5% non-fat milk (Carnation) or 3% bovine serum albumin (BSA; EMD Millipore), followed by probing with primary and secondary antibodies (Table 2). Immunoreactive bands were visualized using either Millipore Immobilon Forte Western HRP Substrate solution or BioRad Clarity Max Western ECL Substrate solution and imaged using a BioRad ChemiDoc XRS+ Imaging System. Resulting bands were analyzed using BioRad Image Lab™ Software, and band intensities of target hepatic mitochondrial proteins were normalized to those of β-Actin as previously published (Wei *et al.* 2009, Zhao *et al.* 2019). Note that while a sample size of 7–8 offspring per group was used for all experiments, representative immunoblots containing all experimental groups on one blot are presented with either 4 offspring (PND 130) or 5–6 offspring (PND 21) per group.

**Table 2:** Western blot primary and secondary antibodies, dilutions and company/catalog information.

Antibody name	Source	Dilution	Company (catalogue no.)
SHC1	Mouse monoclonal	1:1000	Acris Antibodies, Rockville, MD, USA (AM00143PU-N)
Pin1 (G-8)	Mouse monoclonal	1:1000	Santa Cruz Biotechnology Inc., Santa Cruz, CA, USA (sc-46660)
4-Hydroxynonenal	Mouse monoclonal	1:1000	R&D Systems, Oakville, ON, Canada (MAB3249)
Superoxide dismutase (SOD)-1 (FL-154)	Rabbit polyclonal	1:1000	Santa Cruz Biotechnology Inc., Santa Cruz, CA, USA (sc-11407)
Superoxide dismutase (SOD)-2 (FL-222)	Rabbit polyclonal	1:1000	Santa Cruz Biotechnology Inc., Santa Cruz, CA, USA (sc-30080)
Catalase (H-300)	Rabbit polyclonal	1:1000	Santa Cruz Biotechnology Inc., Santa Cruz, CA, USA (sc-50508)
pSer(232) pyruvate dehydrogenase	Rabbit polyclonal	1:1000	EMD Millipore, Etobicoke, ON, Canada (AP1063)
Pyruvate dehydrogenase	Rabbit polyclonal	1:1000	Cell Signaling Technology Inc., Danvers, MA, USA (2784S)
LDHa	Rabbit polyclonal	1:1000	Cell Signaling Technology Inc., Danvers, MA, USA (2012S)
Citrate synthase	Rabbit polyclonal	1:1000	Provided by Dr S. Raha, McMaster University
OXPHOS rodent cocktail	Mouse monoclonal	1:1000	Abcam Inc., Toronto, ON, Canada (ab110413)
TFAM (D5C8)	Rabbit monoclonal	1:1000	Cell Signaling Technology Inc., Danvers, MA, USA (8076S)
KDEL ER marker (10C3)	Mouse monoclonal	1:250	Santa Cruz Biotechnology Inc., Santa Cruz, CA, USA (sc-58774)
β-Actin peroxidase	Mouse monoclonal	1:25,000	Sigma Aldrich Co., St. Louis, MO, USA (A3854-200UL)
Goat anti-rabbit IgG HRP-linked (H + L chain)	N/A	1:10,000	Cell Signaling Technology Inc., Danvers, MA, USA (7074P2)
Horse anti-mouse IgG HRP-linked (H + L chain)	N/A	1:10,000	Cell Signaling Technology Inc., Danvers, MA, USA (7076S)

### Statistical analysis

All statistical analyses were performed using GraphPad Prism 8 software. Results were expressed as means of normalized values ± s.e.m., and the threshold for statistical significance was set as *P* < 0.05. As mentioned previously, a sample size of 7–8 offspring (i.e., litter is the statistical unit) was used for all *in vivo* experiments, as this provided enough statistical power to detect significant differences in outcome measures. Again, immunoblots presented are representative and do not contain all offspring included in statistical analyses, but are rather presented such that all groups are contained on the same blot. To ensure that trends were consistent within the control group on blots used for analysis, data were normalized such that the same control sample was used as an internal loading control (i.e., the first control sample, present in lane one). Results obtained from body weight or qRT-PCR were analyzed using one-way ANOVA followed by Tukey’s multiple comparisons test. Because we were interested solely in the differences between the control diet and each individual MPR treatment (i.e., the timing of nutritional intervention), individual Student’s unpaired *t*-tests were performed on distinct blots comparing each MPR group to their respective control. All cell culture experiments were performed in biological replicates of 3, where each replicate represents an independent experiment using a different frozen cell stock or passage number. These *in vitro* data were analyzed at each time point using a Student’s unpaired *t*-test, as the purpose of this experiment was to determine if tunicamycin-induced ER stress led to elevated p66Shc abundance rather than the effects observed with time.

## Results

### Restoration of dietary proteins following maternal protein restriction results in rapid postnatal catch-up growth

We and others have previously shown that MPR causes decreased birth weight, while the restoration of dietary proteins produces rapid postnatal catch-up growth in the affected offspring (Hales *et al.* 1996, Desai & Hales 1997, Sohi *et al.* 2011). To determine if MPR offspring used in our study underwent whole body and/or liver catch-up growth, body weights and liver weights were recorded at birth and PND 21 and 130 (i.e., 3 weeks and 4 months of age). Using these weights, liver to body weight ratio was calculated for offspring at each time point. At postnatal day 21, LP1/LP2 offspring had significantly decreased average bodyweight and liver weight relative to control offspring (Table 3; *P* < 0.0001), while there were no differences in body weight and liver weight between LP3 and control offspring. Interestingly, liver to body weight ratios of both LP1/LP2 and LP3 offspring were significantly lower than those of control offspring at this time point (Table 3; *P* < 0.01 and *P* < 0.05, respectively). At 4 months of age, LP1 offspring weighed significantly less than control offspring (Table 3; *P* < 0.05), while body weights of LP2 and LP3 offspring were not significantly different from those of control offspring. There were no significant differences between liver weights and liver to body weight ratios of control offspring compared to all groups of MPR offspring at 4 months of age (Table 3).

**Table 3:** Maternal protein restriction followed by restoration of dietary protein leads to rapid postnatal catch-up growth by 4 months of age.

	d21	d130
Body weight (g)		
Control	48.75 ± 1.06^*^	544.25 ± 10.30^†‡^
LP1	26 ± 0.53^†^	467 ± 15.82^*^
LP2	N/A	504.38 ± 11.35^*†^
LP3	46.14 ± 2.97^*^	602 ± 22.12^‡^
Liver weight (g)		
Control	1.91 ± 0.07^*^	17.16 ± 1.84^*^
LP1	0.86 ± 0.001^†^	15.60 ± 0.84^*^
LP2	N/A	16.94 ± 0.79^*^
LP3	1.65 ± 0.002^*^	19.30 ± 1.54^*^
Liver to body weight ratio		
Control	0.04 ± 0.001^*^	0.031 ± 0.003^*^
LP1	0.03 ± 0.001^†^	0.033 ± 0.001^*^
LP2	N/A	0.033 ± 0.001^*^
LP3	0.04 ± 0.001^†^	0.032 ± 0.002^*^

For offspring at both timepoints, liver growth was assessed by calculating liver to body weight ratio. All data are expressed as means ± s.e.m., and dietary effects were determined using one-way ANOVA followed by Tukey’s multiple comparisons test. Groups labeled with different symbols (*, †, ‡) are significantly different from each other.

### Maternal protein restriction during the perinatal period leads to increased hepatic p66Shc following postnatal catch-up growth

Given the strong association between p66Shc and impaired mitochondrial function (Migliaccio *et al.* 1999, Orsini *et al.* 2004, Giorgio *et al.* 2005, Pinton *et al.* 2007, Trinei *et al.* 2013), we first measured the expression levels of hepatic p66Shc in our MPR offspring. While the steady-state mRNA levels of *p66Shc* remained unaffected in all MPR offspring (Fig. 1B), LP2 offspring demonstrated a significant increase in p66Shc protein abundance at 4 months of age compared to control offspring (Fig. 1E; *P* < 0.05). Conversely, p66Shc protein levels were significantly decreased in LP1 and LP3 offspring when compared against that of control offspring (Fig. 1E; *P* < 0.05). These data were consistent with changes in protein abundance of peptidyl-prolyl cis-trans isomerase NIMA-interacting 1 (PIN1), an isomerase that is essential for translocation of p66Shc into mitochondria. Transcript abundance of *Pin1* remained unchanged across all groups (Fig. 1C); however, PIN1 protein abundance was significantly increased in LP2 offspring at 4 months (Fig. 1F; *P* < 0.05). PIN1 protein abundance was not significantly affected in LP1 or LP3 offspring at this time point (Fig. 1F).

### Maternal protein restriction causes increased oxidative stress following postnatal catch-up growth

**Figure 2:**
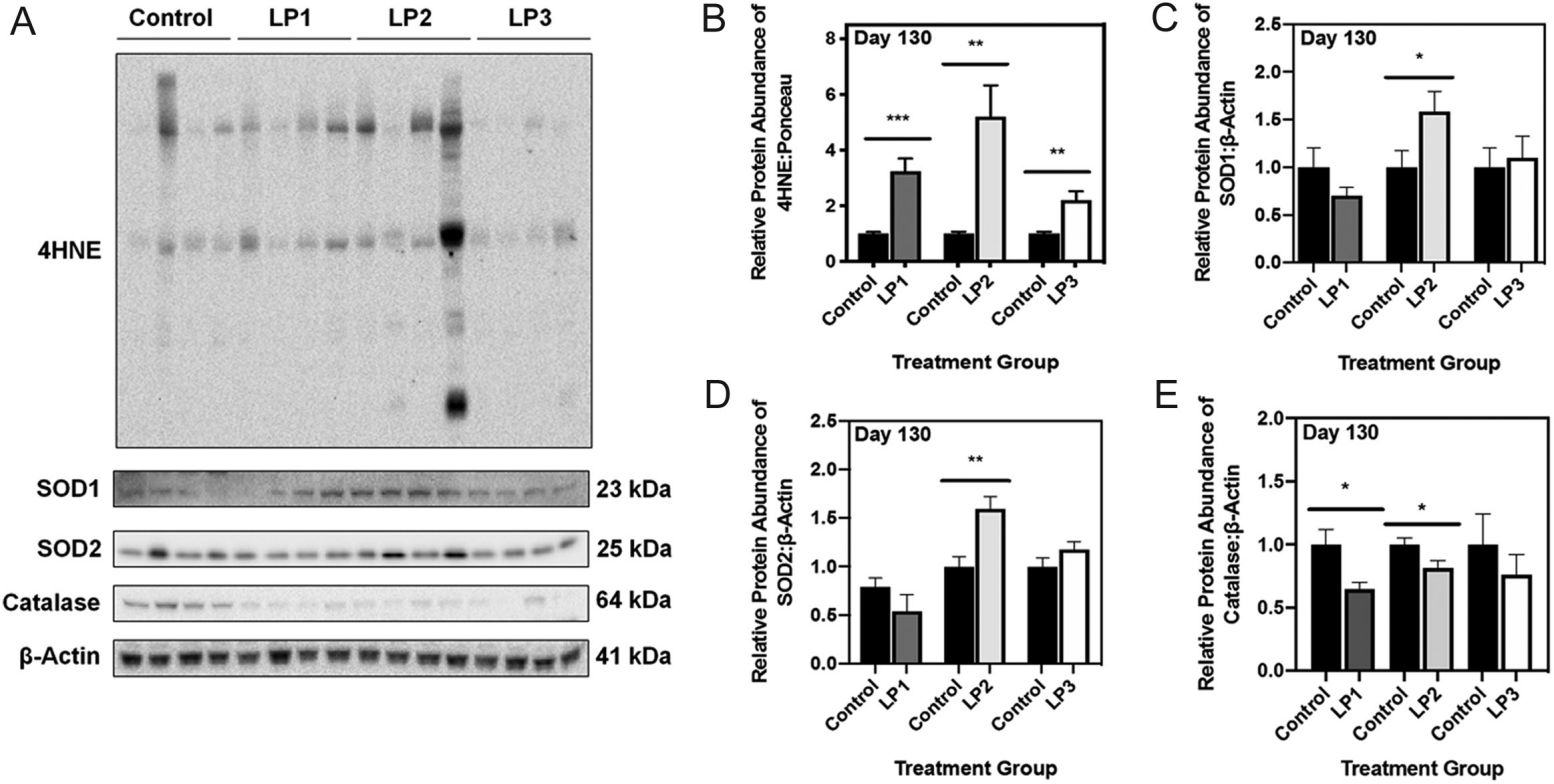
Maternal protein restriction leads to increased markers of hepatic oxidative stress at 4 months of age. (A) Representative Western immunoblots illustrating expression of 4-hydroxynonenal, a marker of lipid peroxidation, superoxide dismutase (SOD)1 and SOD2 in LP1, LP2 and LP3 offspring relative to control diet-fed offspring. Protein abundances of (B) 4HNE, (C) SOD1, (D) SOD2, and (E) catalase in each group of MPR offspring were each compared against control offspring. 4HNE abundance was expressed as means normalized to total protein abundance ± s.e.m. (*n* = 7–8/group), while SOD1, SOD2 and catalase abundance was normalized to β-Actin ± s.e.m. (*n* = 7–8/group). All protein abundances were analyzed using a two-tailed unpaired Student’s *t*-test. *Significant difference (*P* < 0.05), **significant difference (*P* < 0.01), ***significant difference (*P* < 0.001).

Given that increased expression of p66Shc is indicative of oxidative stress, we next measured levels of markers of oxidative stress via Western immunoblotting. When cells exist in a state of oxidative stress, there is increased oxidation of polyunsaturated fatty acids (PUFAs) resulting in the formation of various lipid hydroperoxides. 4-Hydroxynonenal (4-HNE) is among the most bioactive of hydroperoxides produced, and it is recognized as an indirect marker of mitochondrial-induced oxidative stress when present at high levels (Liu *et al.* 2011). Using an antibody specific for 4-HNE adducts of histidine residues, we determined that all groups of 4-month old MPR offspring (LP1, LP2, and LP3) exhibited significantly increased 4-HNE relative to control offspring (Fig. 2B; *P* < 0.001 (LP1) and *P* < 0.01 (LP2 and LP3)). To determine if antioxidant defenses were altered in response to this oxidative stress, we next examined protein abundance of hepatic superoxide dismutase (SOD) 1, SOD2 and catalase. Immunoblot analysis revealed that at four months of age, SOD1 and SOD2 were both significantly increased in LP2 offspring relative to control offspring (Fig. 2C and D; *P* < 0.05 and *P* < 0.01). In contrast, SOD1 and SOD2 protein abundance remained unchanged in LP1 offspring at 4 months of age (Fig. 2C and D; *P* = 0.22 and 0.20), as was the case for LP3 offspring (Fig. 2C and D; *P* = 0.73 and 0.16). Interestingly, abundance of catalase was significantly reduced in both LP1 and LP2 offspring (Fig. 2E; *P* = 0.02 and *P* = 0.03), while LP3 offspring were unaffected (Fig. 2E; *P* = 0.42).

### Maternal protein restriction in combination with postnatal catch-up growth leads to impaired aerobic respiration at 4 months of age

**Figure 3:**
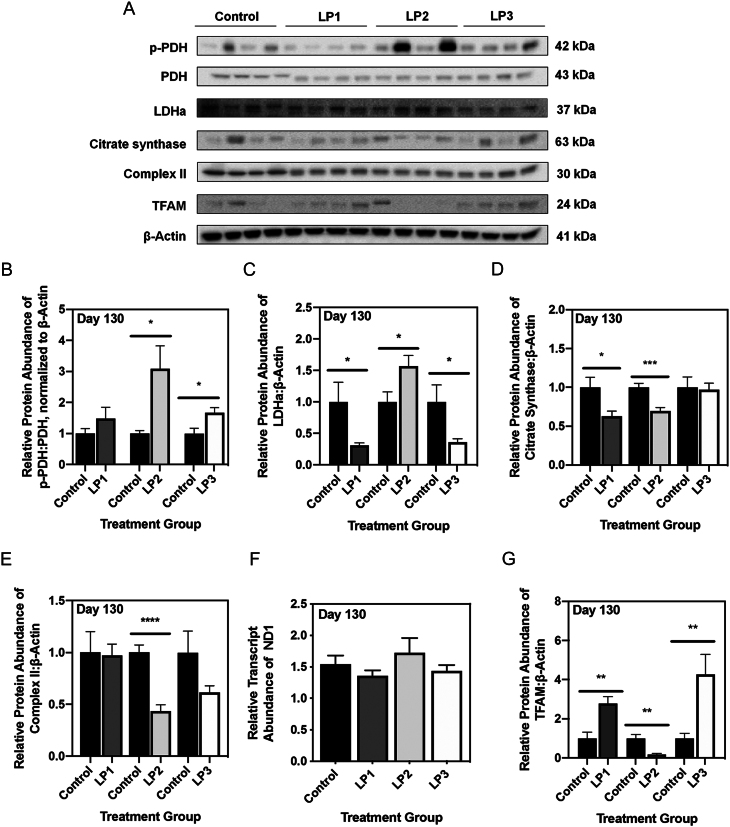
Maternal protein restriction and postnatal catch-up growth together lead to aberrant markers of aerobic metabolism at 4 months of age. (A) Representative Western immunoblots for specific targeted protein bands for control, LP1, LP2 and LP3 offspring as detected by primary antibodies. Protein abundances of (B) phosphorylated pyruvate dehydrogenase (PDH) to total PDH, (C) lactate dehydrogenase subunit A (LDHa), (D) citrate synthase, (E) complex II, and (G) mitochondrial transcription factor A (TFAM) in LP1, LP2 and LP3 offspring were compared against control diet-fed offspring and expressed as means normalized to β-Actin ± s.e.m. (*n* = 7–8/group). (F) Transcript abundance of NADH:ubiquinone oxidoreductase core subunit V1 (ND1), a marker of mitochondrial number, was expressed as means normalized to geometric means of β-Actin and *GAPDH* ± s.e.m. (*n* = 7–8/group). All qRT-PCR data were analyzed using a one-way ANOVA and multiple comparisons test, while protein abundances were compared using a two-tailed unpaired Student’s *t*-test. *Significant difference (*P* < 0.05), **significant difference (*P* < 0.001), ****significant difference (*P* < 0.0001).

Because p66Shc has been suggested to mediate mitochondrial respiration through alterations in oxidative phosphorylation, we next investigated the expression of various enzymes involved in mitochondrial aerobic respiration (Nemoto *et al.* 2006, Acin-Perez *et al.* 2010, Lone *et al.* 2018). Following completion of glycolysis, pyruvate dehydrogenase (PDH) converts pyruvate to acetyl coenzyme-A (acetyl CoA) for use in the citric acid cycle (TCA). Increased phosphorylation of PDH results in inhibited PDH activity, thereby promoting increased glycolysis in the presence of oxygen (i.e., aerobic glycolysis). At 4 months of age, LP2 offspring displayed a significant increase in the ratio of phosphorylated PDH (p-PDH) to total PDH (Fig. 3B; *P* < 0.05), as well as significantly increased levels of lactate dehydrogenase subunit A (LDHa; Fig. 3C; *P* = 0.03). At this time point, the ratio of p-PDH to total PDH was unchanged in LP1 offspring (Fig. 3B; *P* = 0.25), while it was significantly augmented in LP3 offspring (Fig. 3B; *P* < 0.05). Abundance of LDHa was significantly decreased in LP1 and LP3 offspring (Fig. 3C; *P* = 0.02 and *P* = 0.04).

Considering the role of the electron transport chain (ETC) in oxidative phosphorylation, we measured levels of the five protein complexes within the ETC. While protein abundance of complexes I, III, IV and V were unaffected in LP2 offspring (Supplementary Fig. 1B, C, D and E, see section on supplementary materials given at the end of this article), complex II was significantly decreased in LP2 offspring at 4 months of age (Fig. 3E; *P* < 0.0001). In contrast, complex II protein abundance was unaffected in LP1 and LP3 offspring (Fig. 3E). Moreover, the expression levels of mitochondrial complexes I, III, IV and V were also not altered in LP1 and LP3 offspring (Supplementary Fig. 1B, C, D and E).

Given the perinatal environment could have detrimental effects on mitochondrial size and abundance, we first assessed citrate synthase (CS) protein levels as a marker of mitochondrial mass and function. We observed a significant decrease in CS levels of LP2 offspring at 4 months of age relative to control offspring (Fig. 3D; *P* < 0.001). Interestingly, LP1 offspring also displayed a significant decrease in CS levels at this time point when compared against control offspring (Fig. 3D; *P* < 0.05). CS protein levels of LP3 offspring were unaffected compared to control offspring at 4 months of age (Fig. 3D). Since we saw changes in the expression of CS and other mitochondrial metabolic enzymes, we next sought to determine if this was due to changes in mitochondrial number. Using qRT-PCR, we measured hepatic transcript abundance of NADH:ubiquinone oxidoreductase core subunit V1 (ND1) as a marker of mitochondrial number. At 4 months of age, ND1 mRNA abundance was unchanged in all groups of MPR offspring (Fig. 3F). However, immunoblot analysis of mitochondrial transcription factor A (TFAM), which is critical in activating mitochondrial genome transcription, revealed a significant decrease in TFAM abundance in LP2 offspring (Fig. 3G; *P* = 0.004). In contrast, TFAM abundance was significantly increased in LP1 and LP3 offspring (Fig. 3G; *P* = 0.002 and 0.008).

### Maternal protein restriction alone leads to decreased p66Shc protein abundance at 3 weeks of age

**Figure 4:**
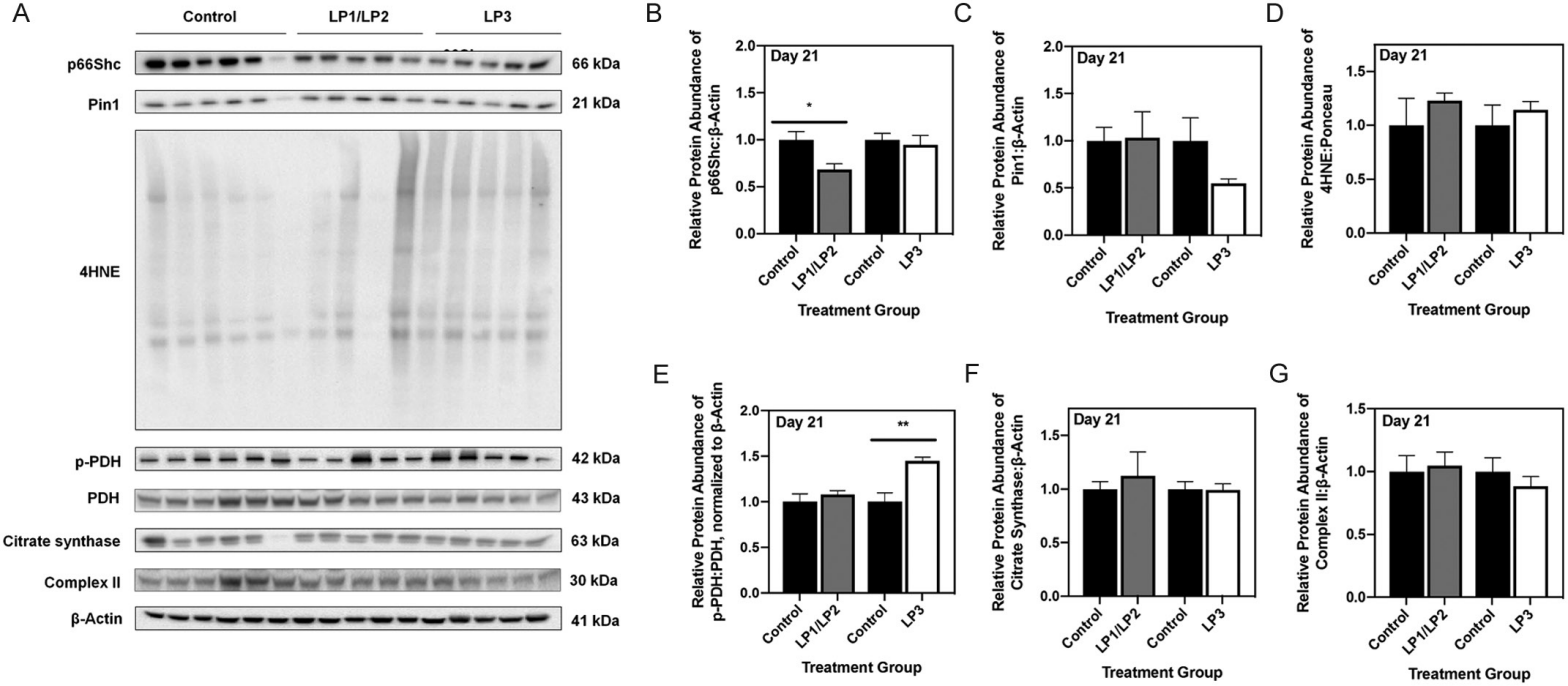
Maternal protein restriction does not independently contribute to increased p66Shc or oxidative stress at 3 weeks of age. (A) Representative Western immunoblots of specific targeted protein bands for control, LP1/LP2, and LP3 offspring as detected by primary antibodies. (B) p66Shc, (C) PIN1, (D) 4HNE, (E) p-PDH, (F) citrate synthase, and (G) complex II protein abundances were compared in each group of MPR offspring against control offspring. 4HNE abundance was expressed as means normalized to total protein abundance ± s.e.m. (*n* = 7–8/group), while abundances of all other targets were normalized to β-Actin ± s.e.m. (*n* = 7–8/group). All protein abundances were analyzed using a two-tailed unpaired Student’s *t*-test. *Significant difference (*P* < 0.05), **significant difference (*P* < 0.01).

In order to distinguish between the direct effects of the MPR diet versus postnatal catch-up growth, we next evaluated the expression of p66Shc and previously examined mitochondrial markers at three weeks (i.e., postnatal day 21). Western immunoblots revealed a significant decrease in p66Shc protein levels in protein-restricted (i.e., LP1/LP2) offspring compared to control offspring (Fig. 4B; *P* < 0.05), while p66Shc was unchanged in LP3 offspring (Fig. 4B). Levels of PIN1, 4HNE, citrate synthase, and all ETC complexes were unaltered in all MPR offspring relative to controls (Fig. 4C, D, F, G and Supplementary Fig. 2B, C, D, E). While the ratio of p-PDH to total PDH was unchanged in LP1/LP2 offspring (Fig. 4E; *P* = 0.44), LP3 offspring displayed a significant increase in p-PDH to total PDH (Fig. 4E; *P* < 0.01).

### Induction of ER stress *in vitro* promotes increased abundance of p66Shc

**Figure 5:**
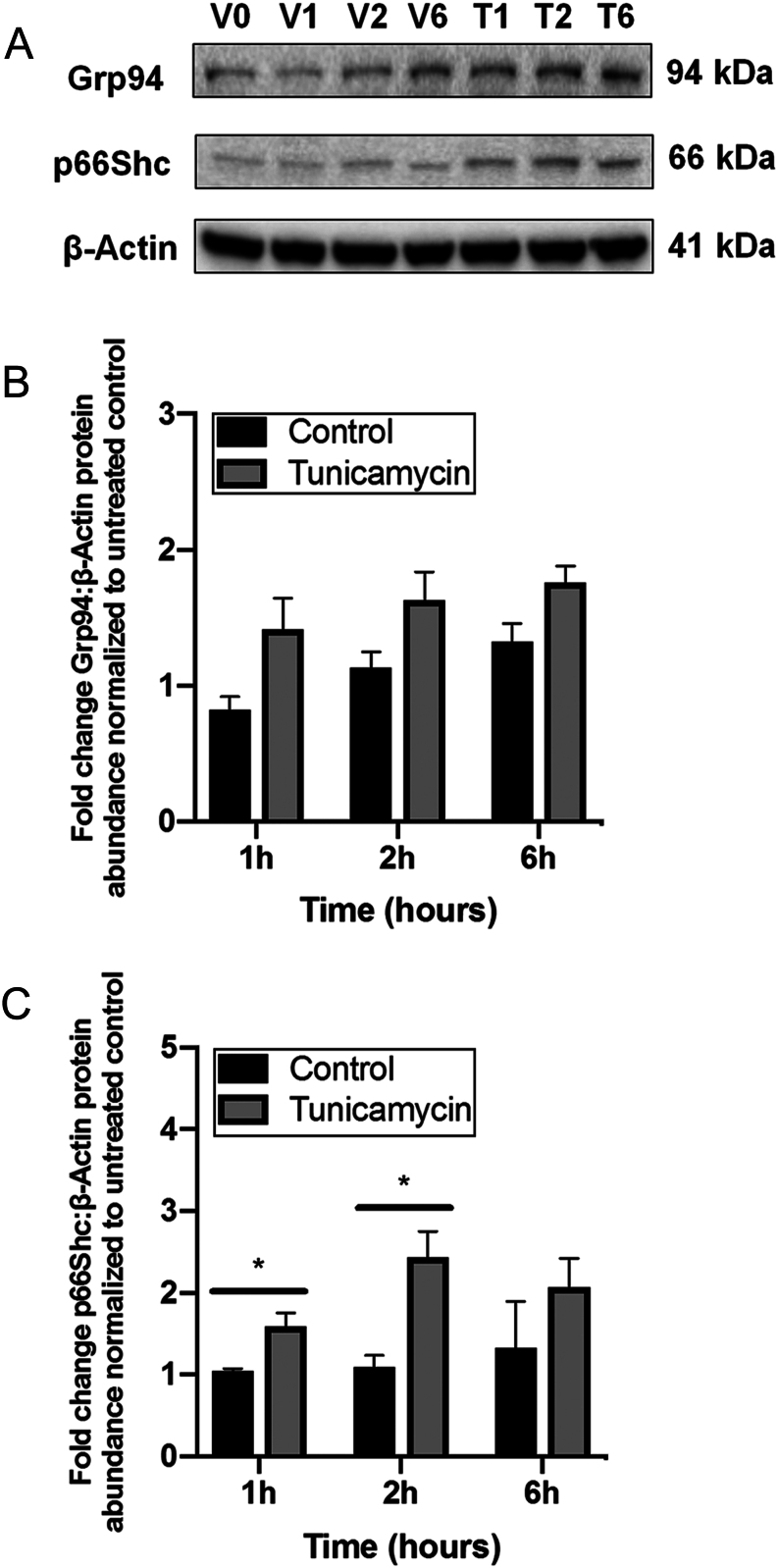
Induction of ER stress with tunicamycin results in increased protein abundance of p66Shc in the HepG2 cell line. (A) Representative Western blots illustrating protein abundance of GRP94 and p66Shc in HepG2 cells treated with either DMSO vehicular control for 1 h (V1), 2 h (V2) or 6 h (V6), or 0.5 µg/mL tunicamycin for 1 h (T1), 2 h (T2) or 6 h (T6). Fold change difference of (B) p66Shc and (C) GRP94 were expressed as means from three biological replicates normalized to β-Actin ± s.e.m., and again normalized to untreated control cells at 0 h (V0). Protein abundances for each time point were analyzed using a two-tailed unpaired Student’s *t*-test. *Significant difference (*P* < 0.05).

Given that our previous studies of these same MPR offspring revealed that only LP2 offspring exhibit ER stress (Sohi *et al.* 2013), and there is an association between increased ER stress and p66Shc (Zhang *et al.* 2013), we next investigated if induction of ER stress *in vitro* leads to augmented hepatic p66Shc protein levels in short-term cell culture. HepG2 cells were used for all *in vitro* experiments given their ability to secrete proteins that are found primarily in fetal hepatocytes (Maruyama *et al.* 2007). Cells were treated with various doses of tunicamycin, a potent inducer of ER stress in HepG2 cells (Lei *et al.* 2016), and collected at 1, 2 and 6 h to determine which dose was most effective in inducing ER stress. A dose of 0.5 µg/mL proved to be most consistent in inducing ER stress, as evidenced by a trending increase in Grp94 protein levels relative to cells treated with DMSO vehicular control (Fig. 5B; *P* = 0.08 (1 h), 0.10 (2 h), 0.07 (6 h)). Interestingly, HepG2 cells treated with tunicamycin had a significant increase in p66Shc protein levels at 1 h and 2 h (Fig. 5C; *P* < 0.05).

## Discussion

**Figure 6:**
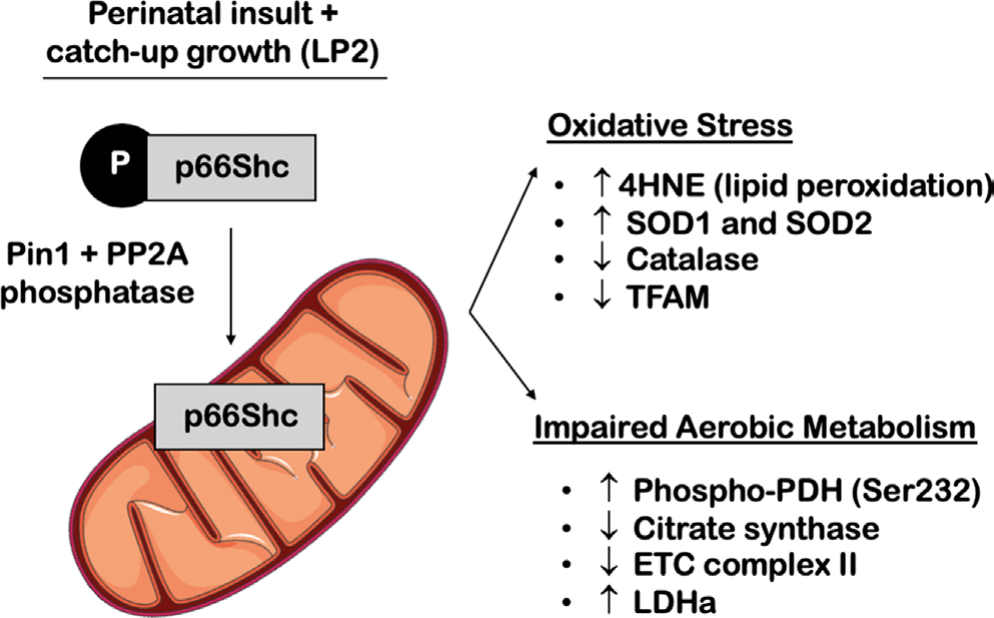
Proposed schematic illustrating the effects of perinatal protein restriction in combination with postnatal catch-up growth on hepatic mitochondrial health. In summary, LP2 offspring were the only experimental group subject to MPR that exhibited increased protein abundance of p66Shc and PIN1. These offspring also displayed aberrant markers of oxidative stress and metabolism, indicating that mitochondrial health had become impaired in adulthood following catch-up growth in early life.

In this study, we demonstrate that perinatal protein restriction with postnatal catch-up growth leads to hepatic oxidative stress and mitochondrial dysfunction in adult rat offspring. Our results suggest that catch-up growth, rather than the direct low protein diet, leads to increased expression of mitochondrial stress marker p66Shc and the cis/trans isomerase PIN1 at 4 months of age. These cellular stresses were exclusive to LP2 offspring, which experienced catch-up growth after completion of liver differentiation (i.e, after 3 weeks) in the rat (Gruppuso & Sanders 2016). It is noteworthy that LP2 offspring are also exposed to the longest period of protein restriction, including 22 days during gestation and 21 days in postnatal life. In addition, LP2 offspring exhibited long-term hepatic oxidative stress, as observed by the apparent changes in expression of 4-HNE, SOD1, SOD2, and catalase. Furthermore, these LP2 offspring display elevated levels of phospho-PDH(Ser232) and LDHa, as well as decreased abundance of CS and mitochondrial ETC complex II, collectively indicating impaired mitochondrial metabolism. Finally, we established a direct relationship between ER stress and hepatic p66Shc expression, thereby uncovering a potential mechanism for increased hepatic p66shc in these LP2 offspring. Taken together, our *in vivo* and *in vitro* results suggest that the dysmetabolism exhibited by MPR offspring is driven in part by mitochondrial dysfunction and oxidative stress (Fig. 6).

While previous studies have verified the significance of oxidative stress and mitochondrial impairment in metabolic disease (Petersen *et al.* 2003, Nojiri *et al.* 2006, Ozgen *et al.* 2012), the role of developmental programming in this process remains largely unknown. In rat offspring, it has been suggested that MPR may be protective during the first month of life by decreasing production of ROS and promoting increased coupling between cellular respiration and oxidative phosphorylation (Moraes *et al.* 2014). Despite this, adult male offspring have been demonstrated to have increased hepatic oxygen consumption and markers of oxidative stress, as well as reduced expression of mitochondrial (mt)-DNA encoded genes (Park *et al.* 2003, They *et al.* 2009, Moraes *et al.* 2014). These data are consistent with human and rodent studies of obesity and cardiovascular disease, as mitochondrial defects arise with morbid obesity and insulin resistance (Dobrian *et al.* 2001, Koliaki *et al.* 2015).

In many cell types, increased oxidative injury is mediated, in part, by augmented expression of p66Shc (Orsini *et al.* 2004, Giorgio *et al.* 2005, Pinton *et al.* 2007). This is particularly evident in the diseased liver, which exhibits elevated expression of p66Shc (Haga *et al.* 2010, Perrini *et al.* 2015, Zhao *et al.* 2019). Using rodent models of various hepatic pathologies, knockdown of p66Shc prevents liver injury by ameliorating oxidative stress and mitochondrial dysfunction (Haga *et al.* 2010, Perrini *et al.* 2015, Zhao *et al.* 2019). Our expression analysis of p66Shc and PIN1 suggests that there may be increased stabilization of p66Shc in LP2 offspring, thereby accelerating hepatic mitochondrial ROS generation. Previous findings demonstrate that MPR offspring with catch-up growth display elevated renal p66Shc much later in life (e.g., 6 months), but this is already a time of significant aging (Luyckx *et al.* 2009). In addition, a comparison to the effects of a low protein cohort (e.g. LP1) were not determined. Conversely, our study highlights how the timing of postnatal nutritional intervention leads to differential expression of hepatic p66Shc expression in young adult offspring. Given that LP1 offspring have decreased expression of p66Shc and unchanged levels of PIN1, it is possible that maintenance of a LP diet in postnatal life may actually protect the adult liver from mitochondrial dysfunction in absence of catch-up growth. Moreover, decreased expression of p66Shc in adult LP3 offspring suggests that introduction of a normal protein diet during lactation, a period of hepatic plasticity, could ‘rescue’ MPR offspring by preventing mitochondrial stress (Gruppuso & Sanders 2016). LP3 offspring undergo catch-up growth prior to the completion of hepatic differentiation (Gruppuso & Sanders 2016); therefore, it is possible that earlier nutritional intervention (i.e. <3 weeks postpartum) allows for recovery from any negative effects of the gestational MPR diet.

Since we observed elevated p66Shc and PIN1 expression in MPR offspring, we next investigated if this coincided with the presence of hepatic oxidative stress. Interestingly, our data revealed that LP2 offspring have increased 4-HNE and SODs, but decreased expression of catalase. Not only does this suggest that LP2 offspring exhibit the greatest degree of oxidative stress, but also that MPR with catch-up growth likely triggers the SOD antioxidant response over time. While this may occur as a compensatory mechanism in response to p66Shc-mediated oxidative stress, it is not sufficient in overcoming the cumulative oxidative stress that is brought about by other mechanisms. That said, decreased abundance of catalase in LP2 offspring may contribute to the apparent oxidative stress via an inability to decompose hydrogen peroxide in the liver. Because 4-HNE, SOD1 and catalase are not specific to the mitochondria, the differential expression of these markers indicates the presence of general cellular oxidative stress as well. That said, SOD2 is localized to the mitochondria (Weisiger & Fridovich 1973); therefore, its upregulation, in combination with increased p66Shc and decreased TFAM, is suggestive of mitochondrial-induced oxidative stress. A previous report indicates that LP2 adult offspring have increased activity of hepatic SOD activity at three months of age; however, direct markers of oxidative stress were not measured (Theys *et al.* 2009). Furthermore, the authors did not examine how the timing of postnatal protein restoration may contribute to this antioxidant response (Theys *et al.* 2009). Collectively, given LP2 offspring are the only MPR group to exhibit abnormal expression of both p66Shc and PIN1 along with all examined markers of oxidative stress, it is conceivable that increased p66shc/PIN1 in these offspring is conducive to oxidative and mitochondrial damage in the liver.

Studies of aging have uncovered the relationship between oxidative stress and impaired mitochondrial metabolism, as oxygen-free radicals can cause inactivation of metabolic enzymes present within the mitochondrion (Cadenas & Davies 2000). More recently, p66Shc has been demonstrated to cause abnormal aerobic respiration (Acin-Perez *et al.* 2010, Lone *et al.* 2018); however, the mechanisms by which this occurs remain relatively unclear. Interestingly, p66Shc stabilization leads to reduced oxygen consumption rate and reduced production of ATP *in vitro* (Lone *et al.* 2018), while its deficiency can reduce basal and maximal oxygen consumption capacity and increase production of lactate by LDHa (Nemoto *et al.* 2006). Moreover, in cultured mouse embryos, greater total p66shc has been correlated with decreased cellular ATP production and increased superoxide production (Edwards *et al.* 2016). Our study determined that MPR and catch-up growth affects all stages of aerobic respiration, as we observed increased phosphorylation of PDH along with decreased abundance of citrate synthase and mitochondrial complex II proteins. Increased p-PDH is indicative of impaired mitochondrial function, as the cell begins to preferentially convert pyruvate to lactate rather than acetyl CoA (Lone *et al.* 2018). This is in agreement with our data concerning LDHa, which was upregulated in adult LP2 offspring. With respect to the ETC, mitochondrial complex II was the only subunit to be influenced by MPR. This is of great interest considering that decreased activity of complex II can cause elevated ROS production within the mitochondria (Walker *et al.* 2006). Citrate synthase, the enzyme responsible for production of citrate from acetyl CoA and oxaloacetate in the TCA, is also a prominent marker of mitochondrial biomass. Decreased hepatic citrate synthase abundance and activity is associated with insulin resistance in male obese rats (Rector *et al.* 2010); therefore, the current decrease in hepatic citrate synthase abundance helps explain the decreased hepatic insulin sensitivity and enhanced gluconeogenesis of LP2 offspring observed in our previous studies (Sohi *et al.* 2013, Vo *et al.* 2013). Finally, the observed decrease in protein levels of TFAM are indicative of reduced mtDNA content. Suppression of TFAM has been previously associated with altered mitochondrial function, suggesting that it may have a role in mediating metabolic activity and disease (Vernochet *et al.* 2012).

To understand why MPR offspring with catch-up growth might exhibit exclusive increases in p66shc, we focused our attention of the role of ER stress on the mitochondria. The ER and mitochondrion are physically connected via sites known as the mitochondrial-associated ER membrane (MAMs), which are highly concentrated with calcium transporters and ion channels (Hayashi *et al.* 2009). These channels can indirectly alter ATP production in response to protein demands of the ER (Burton *et al.* 2017), and it has been demonstrated that their activity is influenced by nutrient availability (Theurey *et al.* 2016). Upon cellular stress, the presence of misfolded and unfolded proteins in the ER initiates the unfolded protein response (UPR), leading to activation of downstream targets that promote an adaptive or apoptotic phenotype (Xu *et al.* 2005). Under severe ER stress, the UPR is initiated to promote the transcription of genes that increase folding capacity of the ER (i.e., Grp94) or protein attenuation (i.e., phosphorylation of eukaryotic initiation factor 2 alpha (eIF2α) (Xu *et al.* 2005). We have previously demonstrated that the same LP2 offspring examined as part of this study exhibit elevated phosphorylated eIF2α and higher expression of chaperone proteins in the liver (Sohi *et al.* 2013). Given that these findings are exclusive to LP2 offspring, it appears that catch-up growth is implicated in onset of hepatic ER stress. Our *in vitro* studies in HepG2 liver cells directly linked ER stress with increased protein abundance of p66Shc, consequently implicating this process as a potential instigator of augmented hepatic p66Shc expression in LP2 offspring. It is noteworthy that in a model of intermittent hypoxia, p66Shc has been associated with increased ER stress in the rat testes; however, the direct role of ER stress on p66Shc was not investigated (Zhang *et al.* 2013). Because we saw an increase in p66Shc as early as 1 h after tunicamycin treatment, as well as an increase only in p66Shc protein but not mRNA *in vivo*, it is possible that ER stress may regulate p66Shc expression via post-transcriptional mechanisms. P66Shc has been demonstrated to be subject to post-transcriptional regulation (Kumar *et al.* 2017); therefore, future *in vitro* studies will investigate this relationship in efforts of elucidating the molecular mechanisms by which ER stress may regulate p66Shc* in vivo*.

In summary, our data indicate that postnatal catch-up growth plays a direct role in the mitochondrial function of adult MPR offspring. We are the first to demonstrate that the timing of nutritional insult can modify expression of hepatic p66Shc, an important modulator of mitochondrial-induced oxidative stress. Protein deficiency exclusively during the perinatal period appears to be most detrimental to hepatic mitochondrial function, while restoration of a normal protein diet at birth shows promise in ameliorating oxidative stress and mitochondrial defects. While these outcomes may be mediated by hepatic ER stress, future studies are warranted in determining the exact mechanism by which this occurs. Overall, our study provides further support for the main tenet of Barker’s ‘thrifty phenotype’ hypothesis, as it highlights the importance of the nutritional environment in developing organ systems and risk for the adult metabolic syndrome.

## Supplementary Material

Supplementary Figure 1. Maternal protein restriction does alter mitochondrial complex proteins I, III, IV and V of the electron transport chain at four months of age. (A) Specific targeted protein bands of control, LP1, LP2, and LP3 offspring as detected by primary antibodies via western immunoblot. (B) Complex I, (C) complex III, (D) complex IV, and (E) complex V protein abundances were each compared in each group of MPR offspring against control offspring. Protein abundances of all targets were normalized to β-Actin ± SEM (n= 7–8/group). All protein abundances were analyzed using a two-tailed unpaired Student’s t-test.

Supplementary Figure 2. Maternal protein restriction does not independently contribute to altered mitochondrial complex proteins I, III, IV and V of the electron transport chain at three weeks of age. (A) Specific targeted protein bands of control, LP1/LP2, and LP3 offspring as detected by primary antibodies via western immunoblot. (B) Complex I, (C) complex III, (D) complex IV, and (E) complex V protein abundances were each compared in each group of MPR offspring against control offspring. Protein abundances of all targets were normalized to β-Actin ± SEM (n= 7–8/group). All protein abundances were analyzed using a two-tailed unpaired Student’s t-test.

## Declaration of interest

The authors declare that there is no conflict of interest that could be perceived as prejudicing the impartiality of the research reported.

## Funding

This work was supported by an NSERC Discovery Grant (RGPIN-04090 to D B H), and a Children’s Health Research Institute Epigenetics Trainee Award to S L O, funded by the Children’s Health Foundation (London, Ontario).

## Author contribution statement

S L O designed the study, performed all experiments and data analysis, and was the primary author in writing the manuscript. G S contributed to study design. D B H assisted in study design and preparation of the manuscript.
