# Supplementary figures and images for: Perinatal protein restriction with postnatal catch-up growth leads to elevated p66Shc and mitochondrial dysfunction in the adult rat liver
Source: Reproduction. 2019 Nov 5;159(1):27–39. doi: 10.1530/REP-19-0188 (PMC6933810; doi:10.1530/REP-19-0188)

**A**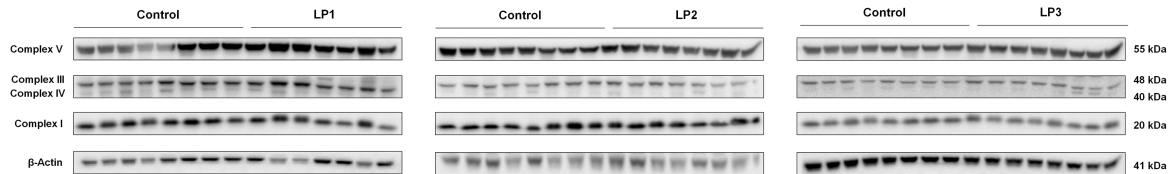**B**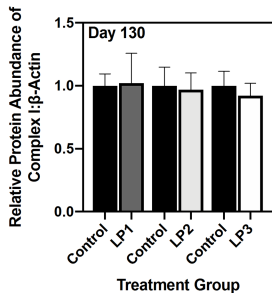**C**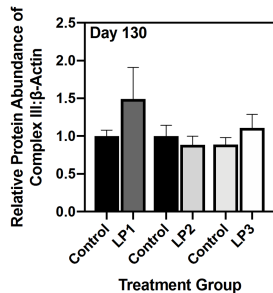**D**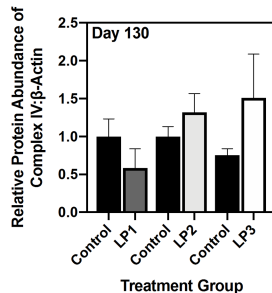**E**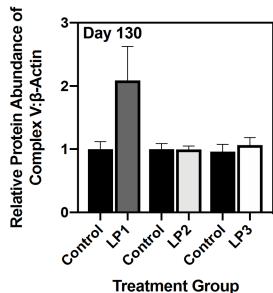

Supplement: Supplementary Figure 1. Maternal protein restriction does alter mitochondrial complex proteins I, III, IV and V of the electron transport chain at four months of age. (A) Specific targeted protein bands of control, LP1, LP2, and LP3 offspring as detected by primary antibodies via western immunoblot. [file supplementary_figure_1.pdf]

**A**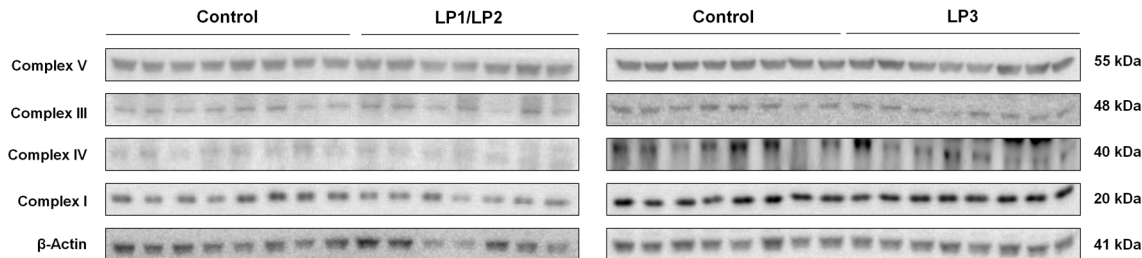**B**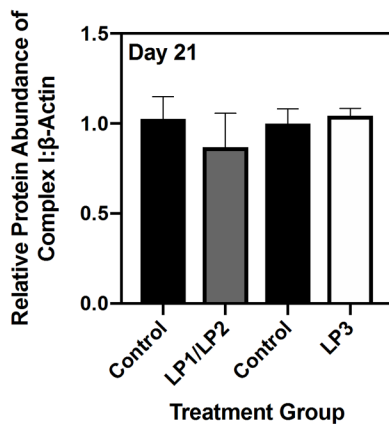**C**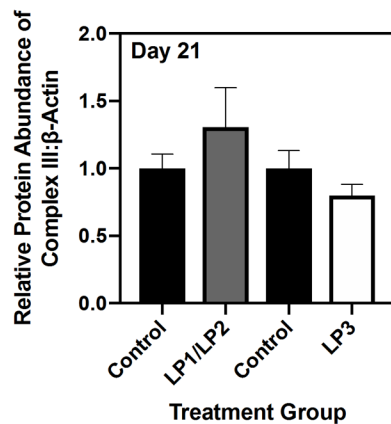**D**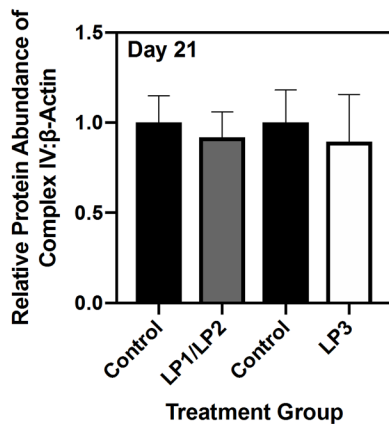**E**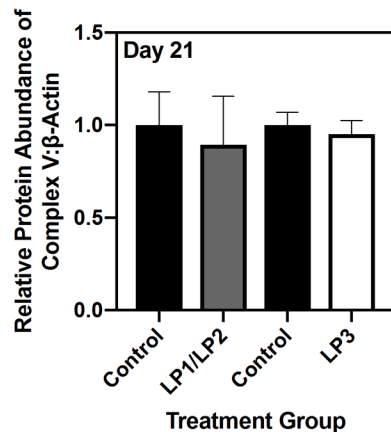

Supplement: Supplementary Figure 2. Maternal protein restriction does not independently contribute to altered mitochondrial complex proteins I, III, IV and V of the electron transport chain at three weeks of age. (A) Specific targeted protein bands of control, LP1/LP2, and LP3 offspring as detected by primary a [file supplementary_figure_2.pdf]
